# Individual and community-level determinants of skin-to-skin contact in Somaliland: a multilevel analysis of the 2020 demographic and health survey data

**DOI:** 10.3389/fgwh.2026.1717086

**Published:** 2026-04-09

**Authors:** Awo Mohamed Kahie, Suhaib Mohamed Kahie, Nura Mohamed Omer, Abdirahman Omer Ali, Nima Muhammad, Saralees Nadarajah

**Affiliations:** 1 School of Postgraduate Studies and Research, Amoud University, Borama, Somalia; 2 College of Health Sciences, School of Medicine and Surgery, Amoud University, Borama, Somalia; 3 Department of Mathematics, University of Manchester, Manchester, United Kingdom

**Keywords:** health facility delivery, maternal health, multilevel analysis, neonatal mortality, newborn care, skin-to-skin contact, Somaliland

## Abstract

**Introduction:**

Neonatal mortality remains a significant public health challenge in Somaliland. Skin-to-skin contact (SSC) is an evidence-based intervention proven to improve newborn survival and maternal health outcomes. However, its implementation in low-resource settings like Somaliland is often limited. This study aimed to determine the prevalence of SSC and identify the individual and community-level factors associated with its practice between mothers and newborns in Somaliland.

**Method:**

This study utilized a cross-sectional design, analyzing secondary data from the 2020 Somaliland Health and Demographic Survey (SLHDS). A weighted sample of 2,139 mothers who had given birth in the preceding three years was included. The primary outcome was the practice of SSC. A multilevel mixed-effects logistic regression model was employed, grouping variables sequentially by geographic/healthcare, individual/child, and socioeconomic categories to account for hierarchical data structure and identify dominant determinants.

**Result:**

The prevalence of mother-to-newborn SSC in Somaliland was 23.8%. The multilevel analysis revealed that both community and individual factors significantly influenced this practice. The most dominant determinant of SSC was the place of delivery; mothers delivering at home had 88% lower odds of practicing SSC compared to those delivering in a health facility (AOR = 0.12, 95% CI: 0.09–0.16). Socioeconomically, mothers from the richest households were 75% more likely to practice SSC than those from the poorest households (AOR = 1.75, 95% CI: 1.17–2.61). Maternal healthcare decision-making capacity was a critical factor: the odds of SSC were significantly lower when the husband decided on healthcare alone (AOR = 0.42, 95% CI: 0.30–0.58) compared to when the mother made decisions independently. Compared to newborns perceived as large at birth, those perceived as small had higher odds of receiving SSC (AOR = 1.88, 95% CI: 1.05–3.37). The null model showed a high intraclass correlation coefficient (ICC = 51.7%), justifying the multilevel approach.

**Conclusion:**

The practice of SSC in Somaliland is alarmingly low, driven primarily by disparities in healthcare access, household wealth, and maternal decision-making autonomy. Future public health strategies should prioritize specific interventions such as subsidized transport to improve facility-based deliveries, mandate evidence-based SSC training for attendants, and implement targeted community campaigns engaging male leaders to empower women in healthcare decision-making.

## Introduction

Skin-to-skin contact (SSC) is defined as placing a naked newborn, covered with a warm blanket, prone on the mother's bare chest (1). Sanabria and Gómez introduced a broader framework encompassing SSC, known as Kangaroo Mother Care (KMC) (2). This practice yields well-documented maternal benefits, including accelerated placental expulsion, reduced postpartum hemorrhage, enhanced breastfeeding self-efficacy, and decreased psychological stress (3). The surge in maternal oxytocin within the first hour after birth is also strongly associated with enhanced mother-infant bonding (4).

Crucially, early SSC acts as an essential catalyst for exclusive breastfeeding (EBF) by fostering the hormonal environment requisite for lactation. Immediate SSC triggers the surge of maternal oxytocin and prolactin, which accelerates milk let-down and stimulates the newborn's innate rooting and suckling reflexes. Consequently, infants who receive early SSC are significantly more likely to initiate breastfeeding successfully and sustain EBF for the recommended first six months of life (5). This successful establishment of EBF has profound implications for the baby's future health condition. In the short term, it provides critical passive immunity against life-threatening neonatal and infant infections, such as sepsis, pneumonia, and severe diarrhea. Looking toward long-term developmental outcomes, infants who receive optimal SSC and subsequent EBF experience a significantly reduced risk of childhood malnutrition, asthma, and chronic non-communicable diseases, including childhood obesity and type 1 diabetes (6). Thus, optimizing SSC is not merely a short-term comfort measure, but an urgent, foundational public health intervention required to secure the long-term physiological stability and health trajectory of children in high-mortality settings. The World Health Organization (WHO) recommends that SSC should begin within the first hour after birth and, under ideal circumstances, can be maintained for up to 23 h a day (7).

The neonatal period—the first 28 days of life—is the most dangerous phase for child survival. Worldwide, approximately 2.5 million child deaths occur in the first month of life, representing 47% of all under-five mortality, primarily in low- and middle-income countries (LMICs). Leading causes of neonatal death include prematurity, birth asphyxia, severe infections, congenital anomalies, and hypothermia. The incidence of neonatal hypothermia can be significantly reduced through early SSC and immediate breastfeeding. Despite these benefits, access to SSC remains limited for both term and preterm infants in LMICs due to healthcare providers’ concerns regarding clinical safety, a lack of standardized training, and systemic institutional barriers (8).

In 2020, over 5.0 million under-five mortalities occurred globally, with newborns accounting for more than 2.4 million of these deaths (9). Epidemiological data demonstrate that approximately two-thirds of neonatal deaths happen within the first 24 h of life. The African region bears the highest neonatal mortality rate (NMR) at 28.0 deaths per 1,000 live births. In East Africa specifically, the NMR stands at 33.6 per 1,000 live births. Expanding access to evidence-based interventions during delivery, including newborn practices like SSC, can substantially reduce mortality associated with preterm birth and neonatal vulnerability (10, 11).

SSC facilitates direct ventral-to-ventral contact, allowing the mother's body to naturally thermoregulate the infant (12). In East Africa, high neonatal mortality is strongly correlated with home births, insufficient maternal education, and healthcare decisions dictated exclusively by husbands (13). Despite its known efficacy, healthcare professionals in many developing regions, including Somaliland, routinely separate mothers and newborns immediately after birth, completely eliminating the opportunity for early maternal contact (14). Reducing the NMR to at least 12 per 1,000 live births by 2030 is a primary target of the UN SDGs. Somaliland faces significant challenges in meeting this goal, with regional estimates showing neonatal mortality near 39 to 42 per 1,000 live births (15).

This study is theoretically grounded in a social-ecological framework, which posits that health behaviors are governed by a complex interplay of individual, community, and structural factors (16–18). This research aims to determine the prevalence of SSC among newborns in Somaliland and identify the specific factors associated with its practice. By utilizing a multilevel analysis structured around distinct categories of determinants, the study explores the hierarchical influences on postpartum behavior, providing actionable evidence to inform targeted public health interventions.

## Methods

### Study area

This study was conducted in Somaliland, an internationally unrecognized self-declared republic located in the Horn of Africa, bordered by Djibouti to the northwest, Ethiopia to the southwest, Somalia to the east, and the Gulf of Aden to the north. Somaliland spans an area of 176,120 square kilometers and experiences a mixed climate characterized by distinct wet and dry seasons. Administratively, the region is divided into six geopolitical zones: Awdal, Marodijeh, Sahil, Togdheer, Sanaag, and Sool. The estimated population is approximately 4.2 million, predominantly consisting of ethnic Somalis who practice Islam. Despite experiencing modest economic growth and relative political stability following its declaration of independence, Somaliland faces severe developmental constraints due to a lack of international recognition, which restricts foreign direct investment and bilateral aid. Pastoralism and livestock export remain the primary economic drivers for both rural and urban communities (19).

### Study design

This research employed a cross-sectional design, utilizing secondary data analysis of the 2020 Somaliland Health and Demographic Survey (SLHDS) dataset.

### Sample size

The original survey included a representative sample of 2,642 respondents. For this analysis, the dataset was cleaned, restricting the sample to mothers who had a live birth in the three years preceding the survey. After applying appropriate sample weights to account for the complex survey design, the final weighted sample size yielded 2,139 mother-infant pairs. This sample encompasses data from all six geographic regions, representing a mix of urban, rural, and nomadic populations.

### Study variables

#### Outcome variable

The primary outcome variable for this study was the practice of skin-to-skin contact between the mother and newborn during the immediate postpartum period. The variable was defined as a binary outcome, derived directly from the SLHDS questionnaire prompt: “After the birth, was (name) placed directly on your bare chest?” Responses were recorded and coded as “1 = Yes” (indicating SSC was practiced) and “0 = No” (indicating SSC was not practiced) (12, 20, 21).

#### Independent variables

The independent variables were conceptually categorized into individual-level and community-level factors based on the social-ecological framework.

**Individual-level variables:** These included maternal age, highest educational attainment, maternal employment status, and exposure to mass media. Media exposure was measured by assessing the frequency of listening to the radio and watching television to determine channel-specific influences. Household characteristics included the sex of the designated household head, the husband's employment status, and the primary healthcare decision-maker in the family. The household wealth index, originally provided as five quintiles in the standard Demographic and Health Survey format, was recoded into three categories (Poor, Middle, Rich). This re-categorization ensured adequate statistical power within each stratum for the multilevel regression models and facilitated a clear interpretation of the socioeconomic gradient. Obstetric characteristics evaluated included the child's sex, the mother's perception of the child's size at birth, birth type (singleton vs. multiple), birth order, preceding birth interval, and whether the pregnancy was planned. Notably, “Place of Delivery” (categorized as Health Facility vs. Home and Others) was designated as an individual-level factor, as it reflects the mother's specific health-seeking behavior and individual service utilization during childbirth.

**Community-level variables:** These variables captured the broader environmental and geographic context, specifically the place of residence (Rural vs. Urban) and the administrative region of residence.

#### Data management and descriptive analysis

Data extraction, variable recoding, and statistical analyses were performed using STATA software, version 16.0. Because the SLHDS utilizes a complex, multi-stage cluster sampling design, all statistical procedures were weighted to ensure sample representativeness and correct for unequal probabilities of selection. The STATA *svy* command set was applied, utilizing individual sample weights (v005), primary sampling unit clustering (v001), and survey stratification variables. Descriptive statistics, presenting weighted frequencies and percentages, were generated to summarize the sociodemographic, economic, and obstetric profiles of the participants, alongside the overall prevalence of SSC.

#### Bivariate and multicollinearity analysis

For the bivariate analysis, a weighted simple logistic regression model was used to evaluate the crude association between each independent variable and the SSC outcome. Variables demonstrating a statistical association with a *p*-value < 0.25 were retained as candidate variables for the multivariable modeling phase. This generous inclusion threshold prevents the premature exclusion of variables that may become significant after adjusting for confounders. Prior to fitting the final models, a multicollinearity diagnostic was conducted across all selected explanatory variables by calculating the Variance Inflation Factor (VIF). All VIF scores were below the standard threshold of 5, confirming the absence of significant multicollinearity. Unadjusted associations are presented as crude odds ratios with corresponding 95% confidence intervals (CIs).

#### Bivariate and multilevel logistic regression modeling

For the bivariate analysis, a weighted simple logistic regression model evaluated the crude association between each independent variable and SSC. Variables with *p* < 0.25 were selected for multivariable modeling.

To account for the hierarchical data structure (mothers nested within communities), a multilevel mixed-effects binary logistic regression model was utilized. To specifically identify the impact of different groups of determinants, a sequential, five-model building strategy was employed:

- **Model 0 (Null Model):** Contained only the outcome variable to decompose total variance and calculate the Intraclass Correlation Coefficient (ICC).
- **Model I:** Adjusted exclusively for Geographic and Healthcare Utilization factors (Region, Place of Delivery).
- **Model II:** Adjusted exclusively for Individual and Child/Obstetric characteristics (Size at Birth, Birth Type, Child's Sex).
- **Model III:** Adjusted exclusively for Socio-Economic and Empowerment factors (Maternal Education, Wealth Status, Husband's Employment, Planned Pregnancy, Health Decision Maker, Media Exposure).
- **Model IV (Full Model):** The final model simultaneously adjusted for all significant factors across Models I, II, and III.

Relative model fit was evaluated using the Log-likelihood Ratio (LR) test, Akaike Information Criterion (AIC), and Bayesian Information Criterion (BIC).

## Results

### Sociodemographic and obstetric characteristics

The study analyzed a weighted sample of 2,139 mothers. Healthcare utilization for delivery was notably low, with 76.1% giving birth at home or in non-clinical settings. The majority of mothers (83.0%) had received no formal education, and over half (52.5%) lived in the poorest wealth stratum (Table 1). Decision-making authority over maternal healthcare was largely controlled by the husband independently (39.8%) or jointly (38.6%).

**Table 1:** Socio-demographic and obstetric characteristics of mothers in Somaliland, 2020 SLHDS (*N* = 2,139).

Variables	Categories	Weighted frequency (*n*)	Percentage (%)
Community-level factors			
Region	Awdal	148	6.90
	Marodijeh	371	17.30
	Sahil	98	4.60
	Togdheer	553	25.88
	Sool	426	19.94
	Sanaag	543	25.38
Place of residence	Rural	1,180	55.17
	Urban	959	44.83
Individual-level factors			
Place of delivery	Health facility	511	23.90
	Home and others	1,628	76.10
Maternal education	No education	1,775	83.00
	Primary	300	14.03
	Secondary	51	2.38
	Higher	13	0.59
Wealth status	Poor	1,123	52.51
	Middle	205	9.56
	Rich	811	37.93
Respondent's age group	15–29 years	1,106	51.69
	30–39 years	910	42.54
	40–49 years	123	5.77
Head of household	Male	1,375	64.30
	Female	764	35.70
Maternal employment	Yes	7	0.33
	No	2,132	99.67
Husband's employment	Yes	912	42.64
	No	1,227	57.36
Listens to radio	At least once a week	98	4.58
	Less than once a week	34	1.60
	Not at all	2,007	93.82
Watches television	At least once a week	234	10.93
	Less than once a week	53	2.46
	Not at all	1,852	86.61
Health decision maker	Respondent	455	21.27
	Husband	852	39.83
	Respondent and husband jointly	826	38.62
	In laws/other	6	0.28
Birth order	1–2	811	37.93
	3	348	16.24
	4+	980	45.83
Birth interval	Less than 2 years	1,255	58.68
	2+ years	884	41.32
Planned pregnancy	Yes	2,065	96.54
	No	74	3.46
Size at birth	Large	288	13.48
	Normal	1,741	81.40
	Small	110	5.12
Birth type	Single	2,081	97.30
	Multiple	58	2.70
Child's sex	Male	1,136	53.10
	Female	1,003	46.90

Frequencies (*n*) and percentages (%) are weighted to account for the complex survey design of the SLHDS. SLHDS, Somaliland Health and Demographic Survey. Due to rounding, percentages may not exactly sum to 100%.

#### Prevalence and bivariate analysis of factors associated with SSC

The weighted prevalence of maternal SSC in Somaliland was 23.8%. The bivariate analysis identified powerful unadjusted associations (Table 2). The place of delivery emerged as an overwhelmingly dominant determinant; SSC was practiced for 63.39% of health facility births compared to only 11.37% of home births (*χ*^2^ = 717.25, *p* < 0.001). Other dominant socio-economic gradients included household wealth (*χ*^2^ = 292.49, *p* < 0.001) and healthcare decision-making autonomy (*χ*^2^ = 100.14, *p* < 0.001).

**Table 2:** Bivariate analysis of factors associated with skin-to-skin contact among mothers in Somaliland.

Variable	Categories	No, *n* (%)	Yes, *n* (%)	*χ*^2^ (*p*-value)
Community-level factors				
Region	Awdal	101 (68.22)	47 (31.78)	*p* < 0.001
	Marodijeh	238 (64.15)	133 (35.85)	
	Sahil	66 (67.05)	32 (32.95)	
	Togdheer	377 (68.21)	176 (31.79)	
	Sool	369 (86.65)	57 (13.35)	
	Sanaag	479 (88.28)	64 (11.72)	
Place of residence	Rural	877 (74.35)	303 (25.65)	*p* = 0.355
	Urban	753 (78.47)	206 (21.53)	
Individual-level factors				
Place of delivery	Health facility	187 (36.61)	324 (63.39)	*p* < 0.001
	Home and others	1,443 (88.63)	185 (11.37)	
Respondent's age group	15–29 Years	855 (77.30)	251 (22.70)	*p* = 0.743
	30–39 Years	682 (74.91)	228 (25.09)	
	40–49 Years	94 (76.12)	29 (23.88)	
Wealth status	Poor	996 (88.76)	127 (11.24)	*p* < 0.001
	Middle	159 (77.60)	46 (22.40)	
	Rich	475 (58.57)	336 (41.43)	
Maternal education	No education	1,413 (79.63)	362 (20.37)	*p* < 0.001
	Primary	191 (63.51)	109 (36.49)	
	Secondary	18 (34.50)	33 (65.50)	
	Higher	8 (64.11)	5 (35.89)	
Sex of household head	Male	1,045 (75.99)	330 (24.01)	*p* = 0.875
	Female	585 (76.57)	179 (23.43)	
Maternal employment	Yes	6 (83.40)	1 (16.60)	*p* = 0.743
	No	1,625 (76.22)	507 (23.78)	
Husband's employment	Yes	576 (63.11)	336 (36.89)	*p* < 0.001
	No	1,054 (85.93)	173 (14.07)	
Health decision maker	Respondent	276 (60.60)	179 (39.40)	*p* < 0.001
	Husband	698 (81.98)	154 (18.02)	
	Jointly	650 (78.73)	176 (21.27)	
	In Laws/Other	6 (100.0)	0 (0.00)	
Listens to radio	At least once a week	81 (82.37)	17 (17.63)	*p* = 0.322
	Less than once a week	20 (60.00)	14 (40.00)	
	Not at all	1,530 (76.24)	477 (23.76)	
Watches TV	At least once a week	122 (52.24)	112 (47.76)	*p* < 0.001
	Less than once a week	13 (24.31)	40 (75.69)	
	Not at all	1,495 (80.70)	357 (19.30)	
Birth order	1–2	616 (75.98)	195 (24.02)	*p* = 0.938
	3	263 (75.56)	85 (24.44)	
	4+	751 (76.60)	229 (23.40)	
Preceding birth interval	Less than 2 years	945 (75.27)	310 (24.73)	*p* = 0.419
	2+ Years	685 (77.51)	199 (22.49)	
Birth type	Single	1,596 (76.69)	485 (23.31)	*p* = 0.102
	Multiple	34 (58.55)	24 (41.45)	
Size at birth	Large	179 (62.26)	109 (37.74)	*p* < 0.001
	Normal	1,386 (79.63)	355 (20.37)	
	Small	65 (58.80)	45 (41.11)	
Planned pregnancy	Yes	1,564 (75.76)	501 (24.24)	*p* = 0.023
	No	66 (88.52)	8 (11.48)	
Child's sex	Male	842 (74.17)	294 (25.83)	*p* = 0.152
	Female	787 (78.50)	216 (21.50)	

The χ^2^ symbol denotes the test statistic obtained from the weighted Pearson Chi-square test, used to evaluate bivariate associations. Variables with a *p*-value < 0.25 were selected for inclusion in the multivariable models.

#### Multilevel analysis of determinants of skin-to-skin contact

The multilevel mixed-effects logistic regression models are presented in Table 3. Grouping variables by their categorical domains revealed deep systemic inequities. In **Model I**, Geographic and Healthcare factors strongly dictated SSC. The place of delivery remained the most formidable predictor; mothers delivering at home had an 88% reduction in the odds of practicing SSC (AOR = 0.12, 95% CI: 0.09–0.16).

**Table 3:** Multilevel logistic regression analysis of grouped determinants of skin-to-skin contact in Somaliland.

Variable	Categories	Model I (Geo/Health) AOR (95% CI)	Model II (Child/Obstetric) AOR (95% CI)	Model III (Socio-Econ) AOR (95% CI)	Model IV (Full Model) AOR (95% CI)
Geographic/healthcare					
Region	Awdal	Ref	-	-	Ref
	Marodijeh	0.60 (0.42–0.87)**	-	-	0.59 (0.40–0.88)**
	Sahil	1.35 (0.92–1.95)	-	-	1.32 (0.91–1.89)
	Togdheer	0.58 (0.39–0.85)**	-	-	0.60 (0.41–0.87)**
	Sool	0.55 (0.39–0.79)**	-	-	0.58 (0.41–0.84)**
	Sanaag	0.58 (0.40–0.84)**	-	-	0.60 (0.41–0.87)**
Place of delivery	Health facility	Ref	-	-	Ref
	Home/others	0.11 (0.08–0.14)***	-	-	0.12 (0.09–0.16)***
Individual/child factors					
Size at birth	Large	-	Ref	-	Ref
	Normal	-	0.77 (0.60–1.01)	-	0.81 (0.61–1.07)
	Small	-	1.55 (1.06–2.28)*	-	1.43 (0.94–2.18)
Child's sex	Male	-	Ref	-	Ref
	Female	-	0.95 (0.78–1.14)	-	0.92 (0.75–1.13)
Birth type	Single	-	Ref	-	Ref
	Multiple	-	1.40 (0.80–2.45)	-	1.15 (0.62–2.12)
Socio-economic factors					
Maternal education	No education	-	-	Ref	Ref
	Primary	-	-	1.40 (1.08–1.82)*	1.34 (1.01–1.77)*
	Secondary	-	-	1.80 (1.05–3.08)*	1.47 (0.85–2.54)
	Higher	-	-	1.65 (0.82–3.30)	1.48 (0.70–3.14)
Wealth status	Poor	-	-	Ref	Ref
	Middle	-	-	1.68 (1.20–2.35)**	1.63 (1.15–2.30)**
	Rich	-	-	1.85 (1.40–2.45)***	1.62 (1.21–2.16)***
Watches TV	At least once/wk	-	-	Ref	Ref
	Less than once/wk	-	-	1.25 (0.68–2.30)	1.36 (0.71–2.62)
	Not at all	-	-	0.65 (0.48–0.88)**	0.76 (0.54–1.07)
Listens to radio	At least once/wk	-	-	Ref	Ref
	Less than once/wk	-	-	2.05 (0.82–5.12)	1.97 (0.75–5.17)
	Not at all	-	-	1.15 (0.72–1.85)	1.20 (0.72–1.98)
Husband's emp.	Yes	-	-	Ref	Ref
	No	-	-	0.65 (0.52–0.81)***	0.72 (0.57–0.91)**
Planned pregnancy	Yes	-	-	Ref	Ref
	No	-	-	0.68 (0.45–1.02)	0.77 (0.49–1.22)
Decision maker	Respondent	-	-	Ref	Ref
	Husband	-	-	0.55 (0.43–0.70)***	0.68 (0.52–0.88)**
	Jointly	-	-	0.52 (0.40–0.66)***	0.60 (0.46–0.78)***
	In laws/others	-	-	1.10 (0.12–9.85)	1.03 (0.10–10.84)

Ref, Reference category; AOR, Adjusted Odds Ratio; CI, Confidence Interval. Asterisks denote statistical significance: * *p* < 0.05, ** *p* < 0.01, *** *p* < 0.001. Model 0 (the Empty Model) is omitted from this table but described in Table 4.

In **Model II**, focused on individual obstetric traits, newborns perceived as small at birth had a higher likelihood of receiving SSC compared to large newborns. The inclusion of birth type (singleton vs. multiple) did not yield statistical significance but was retained based on the bivariate threshold.

**Model III** highlighted the socioeconomic and empowerment dynamics. Household wealth was highly significant, as was the mother's health autonomy. When husbands held sole decision-making authority, the odds of SSC dropped significantly compared to when the mother decided independently.

In the **Full Model (Model IV)**, adjusting for all domains simultaneously, these core determinants maintained their significance. Place of delivery, household wealth quintile, husband's employment, and maternal decision-making power remained the most robust, independent predictors of SSC.

### Random effects analysis and model comparison

Model 0 (the null model) yielded an ICC of 0.517 (*p* < 0.001), indicating that 51.7% of the total variance in SSC probability is attributed to unobserved community differences. The progressive inclusion of specific categorized domains—Geographic/Health (Model I), Individual (Model II), and Socio-Economic (Model III)—explained varying degrees of this variation. The final combined Full Model (Model IV) reduced the ICC to 0.012 and produced the lowest AIC (2,475.44), proving to be the most analytically robust model (Table 4).

**Table 4:** Random effects analysis and model fit statistics for determinants of skin-to-skin contact.

Measurement of variation	Null model (Model 0)	Model I (Geo/Health)	Model II (Obstetric)	Model III (Socio-Econ)	Model IV (Full Model)
*τ* ^2^	3.52	0.55	3.20	1.85	0.04
*p*-value	*p* < 0.001	*p* < 0.001	*p* < 0.001	*p* < 0.001	*p* < 0.001
ICC	0.517	0.143	0.493	0.360	0.012
LR Test	216.78	185.40	198.50	165.20	3.04
Model fit statistics					
AIC	2,236.47	2,580.30	2,895.10	2,610.55	2,475.44
BIC	2,248.23	2,615.40	2,930.25	2,690.40	2,625.29

τ^2^, Community-level variance; ICC, intraclass correlation coefficient. Model IV includes all factors simultaneously.

## Discussion

This study evaluated the prevalence and determinants of maternal SSC in Somaliland utilizing data from the 2020 SLHDS. The national prevalence of SSC was found to be exceptionally low at 23.8%, falling short of both global benchmarks and the East African regional average (51.7%) (22, 23). The low adoption of SSC in LMICs is frequently attributed to a lack of maternal health literacy, entrenched cultural routines, an absence of trained personnel, and systemic socioeconomic constraints (24–27).

Crucially, the bivariate and subsequent multilevel analyses identified specific, overwhelmingly dominant determinants of SSC. The most profound disparity identified was the place of delivery. The bivariate analysis revealed a stark reality: 63.39% of newborns delivered in health facilities received SSC, compared to a mere 11.37% of those delivered at home—a gap reflected in a massive Chi-square value (*χ*^2^ = 717.25, *p* < 0.001). This dominance persisted through the final multilevel model (Model IV), demonstrating that mothers delivering at home had an 88% reduction in the odds of SSC. Health facilities provide the clinical environment necessary to initiate SSC, including trained birth attendants who can safely manage the newborn and enforce clinical guidelines (12, 21, 28, 29).

The infant's biological characteristics played a secondary, though notable, role. Adjusting for confounding variables, mothers of infants perceived as small exhibited a higher likelihood of initiating SSC compared to those with infants perceived as large (30, 31). This suggests that visible physical vulnerability in the newborn acts as a direct motivator for mothers to deploy protective measures, such as thermal regulation via skin contact. No statistically significant difference was found based on the child's sex or birth type, implying that immediate maternal nurturing instincts generally override gender biases in the immediate postpartum window (33–35).

Socio-economic factors and empowerment metrics formed the second most dominant cluster of determinants. High household wealth consistently mitigated barriers to health access and information, heavily favoring SSC initiation (32). However, the most striking finding in this category—and another highly dominant determinant in the bivariate analysis (*χ*^2^ = 100.14)—was the profound impact of maternal decision-making power. When the husband served as the primary or joint decision-maker for maternal healthcare, the probability of the mother practicing SSC fell drastically. In traditional Somaliland households, men typically possess the ultimate authority to sanction health-seeking activities, yet women assume near-exclusive responsibility for physical childcare. This structural disconnect severely restricts a mother's capacity to utilize her own knowledge and instincts during the immediate postpartum period.

### Strengths and limitations of the study

This study benefits from several methodological strengths. It utilized a nationally representative dataset (the SLHDS), facilitating generalizability to the wider population of reproductive-age women in Somaliland. The application of a multilevel mixed-effects model appropriately adjusted for the hierarchical structure of the data, minimizing estimation bias. Furthermore, the rigorous application of sampling weights ensured the results accurately reflect the complex survey design.

However, certain limitations must be acknowledged. The cross-sectional nature of the data restricts the analysis to statistical associations, precluding any definitive conclusions regarding causality. Data regarding SSC practices were self-reported for births occurring up to three years prior to the survey, introducing the potential for recall bias and social desirability bias. The reliance on the mother's subjective perception of the infant's birth size is prone to misclassification compared to objective clinical anthropometry. Lastly, the analysis was limited to the variables collected within the SLHDS. Critical unmeasured factors—such as the attitudes of traditional birth attendants, specific cultural taboos regarding postpartum blood, or the exact duration and quality of the SSC provided—could not be assessed. Residual confounding from these unmeasured variables remains a possibility.

### Implications for policy

The results of this study outline clear policy directives for reducing neonatal mortality in Somaliland. Because facility delivery is the undisputed primary predictor of SSC, specific, targeted interventions are required. The Ministry of Health and international partners must implement subsidized maternal transport voucher programs to overcome the geographic and financial barriers preventing facility access. Additionally, rural health posts must be upgraded to provide 24/7 Basic Emergency Obstetric and Newborn Care (BEmONC).

At the facility level, it is not enough to merely encourage facility deliveries; the quality of care must be standardized. The Ministry should mandate the integration of the WHO's Essential Newborn Care Course (ENCC) and the Helping Babies Breathe (HBB) curriculum into the national training of all midwives and nurses to institutionalize the “Golden Hour” protocols. Finally, because maternal autonomy so heavily dictates health behavior, interventions cannot remain confined to the clinic. Community health programs must launch specific male-involvement campaigns that engage influential religious leaders (Imams) and community elders. These campaigns should actively promote maternal and newborn care as a shared family responsibility rather than a women-only domain, directly targeting patriarchal norms to foster joint or female-led healthcare decision-making.

## Conclusion

SSC practice in Somaliland is alarmingly low, with fewer than one in four newborns receiving this critical intervention. The multilevel analysis, grouped by specific ecological domains, unequivocally demonstrates that SSC is driven by a hierarchy of systemic inequities. Delivery within a formal health facility is the dominant facilitator, while a lack of maternal autonomy in healthcare decision-making acts as a severe barrier. Improving neonatal survival and laying the foundation for exclusive breastfeeding in Somaliland will require specific infrastructural upgrades, including subsidized transport for institutional deliveries, mandatory evidence-based newborn care training for providers, and culturally resonant campaigns that actively involve male leaders to empower women's health autonomy.

## Data Availability

The raw data supporting the conclusions of this article will be made available by the authors, without undue reservation.
